# Effects of Recombinant Human Erythropoietin on Resistance Artery Endothelial Function in Stage 4 Chronic Kidney Disease

**DOI:** 10.1161/JAHA.113.000128

**Published:** 2013-04-24

**Authors:** Marie Briet, Tlili Barhoumi, Muhammad Oneeb Rehman Mian, Cristina Sierra, Pierre Boutouyrie, Michael Davidman, David Bercovitch, Sharon J. Nessim, Gershon Frisch, Pierre Paradis, Mark L. Lipman, Ernesto L. Schiffrin

**Affiliations:** 1 Lady Davis Institute for Medical Research, Sir Mortimer B. Davis‐Jewish General Hospital, McGill University, Montreal, Quebec, Canada (M.B., T.B., M.O.R.M., C.S., P.P., E.L.S.); 2 Department of Medicine, Sir Mortimer B. Davis‐Jewish General Hospital, McGill University, Montreal, Quebec, Canada (M.B., M.D., D.B., S.J.N., G.F., M.L.L., E.L.S.); 3 Department of Pharmacology and Institut National de la Santé et de la Recherche Médicale U970‐PARCC, Hôpital Européen Georges Pompidou, Assistance Publique‐Hôpitaux de Paris, Paris, France (M.B., P.B.)

**Keywords:** chronic kidney disease, endothelial function, erythropoietin, resistance artery

## Abstract

**Background:**

Recent studies have raised concern about the safety of erythropoiesis‐stimulating agents because of evidence of increased risk of hypertension and cardiovascular morbidity and mortality in chronic kidney disease (CKD) patients. In the present study, we investigated the effects of recombinant human erythropoietin (EPO) on endothelial function of gluteal subcutaneous resistance arteries isolated from 17 stage 4 patients (estimated glomerular filtration rate 21.9±7.4 mL/min per 1.73 m^2^) aged 63±13 years.

**Methods and Results:**

Arteries were mounted on a pressurized myograph. EPO impaired endothelium‐dependent relaxation in a concentration‐dependent manner. The maximal response to acetylcholine with EPO at 1, 10, and 20 IU/mL was reduced by 12%, 34%, and 43%, respectively, compared with the absence of EPO (*P*<0.001). EPO‐induced endothelial dysfunction was significantly associated with carotid stiffness and history of cardiovascular events. EPO had no effect on norepinephrine‐induced vasoconstriction or sodium nitroprusside–induced relaxation. ABT‐627, an endothelin type A receptor antagonist, and tempol, a superoxide dismutase mimetic, partially reversed the altered endothelial function in the presence of EPO (*P*<0.01). Increased expression of endothelin‐1 was found in the vessel wall after incubation with EPO.

**Conclusions:**

EPO alters endothelial function of resistance arteries in CKD patients via a mechanism involving in part oxidative stress and signaling through an endothelin type A receptor. EPO‐induced endothelial dysfunction could contribute to deleterious effects of EPO described in large interventional trials.

## Introduction

Erythropoiesis‐stimulating agents were approved 20 years ago for the treatment of anemia associated with chronic kidney disease (CKD) and have been widely accepted for this indication. However, large clinical trials raised concerns about their safety, especially in high‐hemoglobin target groups.^1^ The Normal Hematocrit Study involving patients with congestive heart failure or ischemic heart disease who were receiving hemodialysis was stopped because an interim analysis showed an excess of deaths and nonfatal myocardial infarction in the high‐hematocrit group.^2^ Clinical trials including patients with stage 3 to 4 CKD also showed an increase in cardiovascular (CV) events^3–4^ and hypertension^5^ in the highest‐hemoglobin group. Whether the increase in CV events was due to hematocrit levels or the dosage of erythropoiesis‐stimulating agents required to reach the hematocrit target is still controversial.

Experimental models suggest that EPO influences nitric oxide (NO) production and endothelial function. In human endothelial cell lines, EPO suppresses endothelial NO synthase expression and NO production.^6^ In rodent models, EPO has been shown to exert either protective^7–8^ or deleterious^9^ effects on endothelial function depending on the rodent model and the size of the studied artery.^6–8^ EPO‐induced hypertension in patients and animal models with CKD has been associated with higher endothelin (ET)‐1 plasma levels.^10^ In bovine pulmonary artery endothelial cells, EPO induced ET‐1 secretion in a dose‐dependent manner.^11^ ET‐1 has been shown to induce endothelial dysfunction in, for example, mice overexpressing ET‐1^12^ or in the deoxycorticosterone acetate–salt hypertensive rat model.^13^ Taken together, the data obtained in rodents and cell lines suggest that EPO‐induced vascular injury could involve ET‐1 and endothelial dysfunction. However, in humans, and particularly in CKD patients, the effect of EPO on small resistance arteries has never been studied, and neither has the potential involvement of ET‐1.

Resistance artery remodeling and endothelial dysfunction have been associated with various diseases, including hypertension,^14^ obesity,^15^ and diabetes,^16^ and have a negative impact on patient outcomes.^17–18^ Resistance arteries isolated from biopsy samples of gluteal subcutaneous tissue provide a unique opportunity to evaluate the function of human resistance arteries and to test the direct effects of pharmacological agents.^19^ We hypothesized that human recombinant EPO alters endothelial function of subcutaneous resistance arteries isolated from CKD patients through induction of ET‐1 expression and oxidative stress. We directly tested the effect of various concentrations of EPO on endothelial function of these vessels and the potential preventive effect of an ET type A receptor (ET_A_R) antagonist and of antioxidant pretreatment.

## Methods

### Patients

The study protocol was approved by the Human Research Ethics Review Committee of the Jewish General Hospital, where the study was carried out. All the patients included in the study provided written informed consent to participate. From March 2010 to December 2010, 17 patients with stage 4 CKD were included in the study on the basis of an estimated glomerular filtration rate between 15 and 30 mL/min per 1.73 m^2^ (Modification of Diet in Renal Disease equation).^20^ Patients enrolled were 18 years of age or older, had not been on dialysis, and had not received a kidney transplant. None of the patients had previously received an erythropoiesis‐stimulating agent. Pregnant women were excluded. Diabetic nephropathy was identified in 6 patients, nephroangiosclerosis in 4 patients, immunoglobulin A nephropathy in 4 patients, tubulointerstitial nephropathy in 1 patient, and undetermined nephropathy in 2 patients.

### Large Artery Evaluation

All patients were studied in a quiet room with controlled temperature of 22°C dedicated to arterial measurements. Brachial blood pressure was measured with use of an automated device (BPM‐300; BpTRU Medical Devices) after a 5‐minute rest in the sitting position in the absence of observers 6 times at 1‐minute intervals, and the mean of the last 5 measurements was used. End‐diastolic internal diameter, stroke change in diameter, and intima‐media thickness were measured on the right common carotid artery 2 cm before the bifurcation with a high‐precision echotracking device (ArtLab System; Esaote), as previously described and validated.^21^ Arterial pressure waveforms were measured at the level of the right radial artery and the common carotid artery with applanation tonometry (SphygmoCor; Atcor Medical), as previously validated.^22–24^ Carotid distensibility was determined from systolic–diastolic variations in arterial cross‐sectional area (ΔA) and carotid pulse pressure (ΔP), assuming the lumen to be circular. The cross‐sectional distensibility coefficient (DC) was calculated as ΔA/AΔP. Carotid stiffness was calculated as DC^½^ as previously described.^24^ Aortic stiffness was measured as the carotid‐to‐femoral pulse wave velocity according to the foot‐to‐foot velocity method (SphygmoCor). Pulse transit time (seconds) between the carotid and femoral sites was divided by the distance (m) to obtain carotid‐femoral pulse wave velocity (m/s).^24–25^

### Subcutaneous Resistance Artery Studies

Gluteal subcutaneous biopsies were performed under local anesthesia (2% lidocaine). Small arteries (lumen diameter 150 to 300 μm) were isolated and mounted on a pressurized myograph as previously described and validated.^26–27^ Briefly, the vessels were equilibrated for 60 minutes at 60 mm Hg intraluminal pressure in warmed Krebs solution (pH 7.4) containing (in mmol/L): NaCl 120, NaHCO_3_ 25, KCl 4.7, KH_2_PO_4_ 1.18, MgSO_4_ 1.18, CaCl_2_ 2.5, EDTA 0.026, and glucose 5.5, bubbled continuously with 95% air and 5% CO_2_ at 37°C. Small arteries were preincubated for 15 minutes with vehicle (polysorbate‐80, 0.3 mg/mL; Sigma Aldrich, concentration recommended by the manufacturer) or EPO (epoietin‐α [Eprex]; Janssen‐Cilag) at the indicated concentrations before each curve. Endothelium‐dependent relaxation was assessed by measuring the dilatory response to acetylcholine (10^−9^ to 10^−4^ mol/L) in norepinephrine‐precontracted vessels (10^−6^ mol/L) in the presence of increasing EPO concentrations (1, 10, and 20 IU/mL). Concentration‐response curves to acetylcholine (10^−9^ to 10^−4^ mol/L) in the presence of EPO (20 IU/mL) were repeated after preincubation (15 minutes) with and in the presence of tempol (10^−3^ mol/L; Sigma Aldrich), a superoxide dismutase mimetic, and ABT‐627 (10^−7^ mol/L; Abbott Laboratories), a selective ET_A_R antagonist. Endothelium‐independent relaxation was assessed with sodium nitroprusside (10^−8^ to 10^−3^ mol/L) in norepinephrine (10^−6^ mol/L)‐precontracted vessels in the presence and absence of EPO (20 IU/mL). Concentration‐response curves to norepinephrine (10^−8^ to 10^−4^ mol/L) were performed in the presence and absence of EPO (20 IU/mL).

### Immunohistochemistry Staining

ET‐1 expression in subcutaneous resistance arteries was evaluated by immunohistochemistry. Arteries were fixed in 4% paraformaldehyde for 20 minutes on the myograph at a constant pressure of 60 mm Hg. The artery was transferred to a tube containing the fixative solution, and the fixation was continued for 48 hours with gentle agitation at 4°C. Five‐micrometer paraffin‐embedded sections were deparaffinized, rehydrated, and washed with Tris‐buffered saline (TBS). Thereafter, sections were incubated with a blocking solution (10% normal goat serum [NGS] solution made up in TBT [TBS supplemented with 0.1% Tween‐20]) for 30 minutes, followed by incubations for 15 minutes each with streptavidin and biotin solutions (Vector Laboratories). Sections were incubated with mouse monoclonal anti–ET‐1 antibody (1:50; Sigma Aldrich) in blocking solution overnight at 4°C. Sections were washed with TBT and incubated for 30 minutes with 0.3% H_2_O_2_ to quench endogenous peroxidase and washed with water, TBS, and TBT. Sections were then incubated for 1 hour with blocking solution at room temperature (RT) followed with a biotinylated horse antimouse antibody (1:250; Vector Laboratories) for 1 hour at RT. Sections were washed with TBT and incubated for 1 hour with blocking solution at RT and then incubated with streptavidin‐HRP reagent (1:2000; PerkinElmer) in blocking solution for 30 minutes and washed with TBT. The sections were incubated with a solution containing 0.05% 3,3′‐diaminobenzidine tetrahydrochloride (Sigma Aldrich) and 0.03% H_2_O_2_ in TBS for 4 minutes to reveal the conjugate complexes and washed with water to stop the reaction. Finally, the sections were counterstained with methyl green for 1 minute, dehydrated, and mounted with Eukitt mounting reagent (Calibrated Instruments, Inc). Images were captured with Norton Eclipse software.

### Immunofluorescence Staining

Five‐micrometer paraffin‐embedded sections were deparaffinized with xylene, rehydrated using solutions of decreasing concentrations of ethanol (100% to 50%), and transferred to phosphate‐buffered saline (PBS). Thereafter, sections were incubated with a blocking solution (10% NGS solution made up in PBST [PBS 1× supplemented with 0.1% Tween‐20]) for 1 hour at RT. Sections were incubated with a rabbit antihuman ETAR antibody (1:500; Abcam Inc) in blocking solution overnight at 4°C. The sections were washed 3 times with PBST. Sections were incubated with Alexa Fluor 647 goat antirabbit antibody (1:200; Invitrogen Corp) in blocking solution for 1 hour at RT and then washed 3 times with PBST. Sections were stained with 3 μmol/L 4′,6‐diamidino‐2‐phenylindole (DAPI, Invitrogen) in PBS, washed 3 times with PBS, and mounted in Fluoromount (Sigma‐Aldrich Canada). Images were captured using a fluorescent microscope Leica DM2000 (Leica Microsystems) and analyzed with use of ImageJ.

### Biological Parameters

Blood and urine were sampled the day of patient inclusion in the morning under fasting conditions. Blood was collected in EDTA and heparin tubes. Hemoglobin, plasma creatinine, triglycerides, high‐ and low‐density lipoprotein cholesterol, and urinary albumin and creatinine were measured in the Department of Diagnostic Medicine at the Jewish General Hospital according to routine methods. The estimated glomerular filtration rate was determined by the Modification of Diet in Renal Disease formula.^20^

### Statistical Analyses

Data are expressed as mean±SD in Table 1. Comparisons between concentration‐response curves were performed by 2‐way ANOVA considering factor group (fixed) and patient (random) using area under the curve followed by a Newman–Keuls post hoc test. Univariate correlation analyses were used to determine which arterial parameter was associated with the alteration of endothelial function with EPO. A nonparametric test (Mann–Whitney *U* test) was performed when the variability was different between groups. All tests were performed using NCSS 2007 software (Gerry Hintze, Kaysville, UT). *P*<0.05 was considered statistically significant.

**Table 1.** Clinical, Biological, and Arterial Characteristics of the 17 Patients Included

Parameters	Mean (SD)
Demographic parameters	
Age, y	63.5 (12.6)
Sex, % male	53
Hypertension, %	100
Dyslipidemia, %	82
Diabetes, %	47
Cardiovascular history, %	23
Calcium channel blockers, %	68
ACE inhibitors, %	19
ARBs, %	62
Diuretics, %	50
β‐Blockers, %	31
Statins, %	75
EPO, %	0
Clinical parameters	
Systolic blood pressure, mm Hg	130 (17)
Diastolic blood pressure, mm Hg	72 (10)
Mean blood pressure, mm Hg	91 (11)
Heart rate, bpm	69 (8)
BMI, kg/m^2^	27.2 (6.3)
Biological parameters	
Creatinine, μmol/L	301 (82)
eGFR, mL/min	21.9 (7.4)
UACR, g/g creatinine	0.33 (0.16)
Hemoglobin, g/dL	109 (13.1)
Total cholesterol, mmol/L	4.1 (1.7)
HDL cholesterol, mmol/L	1.2 (0.7)
LDL cholesterol, mmol/L	1.9 (1.1)
Triglycerides, mmol/L	2 (1.2)
Large artery function	
Carotid‐femoral pulse wave velocity, m/s	13.0 (3.4)
Carotid intima‐media thickness, μm	607 (143)
Carotid stiffness, m/s	7.3 (1.7)

Values are mean±SD or % of subjects (N=17). ACE indicates angiotensin‐converting enzyme; ARB, angiotensin receptor blocker; EPO, recombinant human erythropoietin; BMI, body mass index; eGFR, estimated glomerular filtration rate; UACR, urinary albumin/creatinine ratio; HDL, high‐density lipoprotein; LDL, low‐density lipoprotein.

## Results

### Studied Population

Clinical, biological, and arterial characteristics are depicted in Table 1. Seventeen patients with stage 4 CKD (estimated glomerular filtration rate 21.9±7.4 mL/min per 1.73 m^2^) with controlled hypertension who were aged 63.5±13 years and had not been treated with EPO were included in the study. Approximately half the patients were diabetic and overweight, and 82% exhibited dyslipidemia. Patients were treated mainly with renin–angiotensin system blockers, calcium channel blockers, and statins. Compared with previous published data^23–24^ on CKD stage 3 and 4, the population studied had comparable values of carotid stiffness, higher carotid‐femoral pulse wave velocity, and lower values of carotid intima‐media thickness.

### Vasoactive Effects of EPO on Subcutaneous Small Arteries

**Figure 1.**
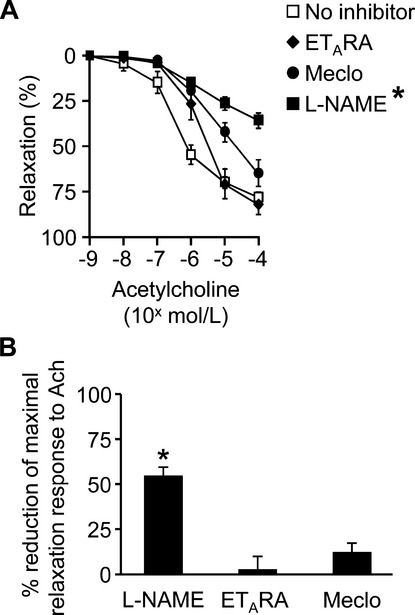
Endothelial function of subcutaneous resistance arteries in chronic kidney disease patients. The vasodilatory response to acetylcholine (Ach) of resistance arteries isolated from chronic kidney disease patients is mostly nitric oxide synthase‐dependent. A, Vasodilatory concentration‐response curves to acetylcholine were obtained without (no inhibitor) and after preincubation (15 minutes) with the nitric oxide synthase inhibitor *N*^ω^‐nitro‐l‐arginine methyl ester (l‐NAME, 10^−4^ mol/L) in 15 patients, the cyclooxygenase inhibitor meclofenamate (meclo, 10^−6^ mol/L) in 4 patients, or the endothelin‐1 receptor type A blocker ABT‐627 (ET_A_RA, 10^−7^ mol/L in 6 patients. The percent reduction of the maximal relaxation response to Ach is shown in (B). *P<0.01 vs no inhibitor.

To determine whether EPO alters endothelial function through a direct action on the vasculature independent of hemodynamic effects, subcutaneous resistance arteries were isolated from CKD patients and endothelium‐dependent relaxation responses to acetylcholine were determined without and with EPO. In the absence of EPO, the maximal relaxation to acetylcholine was 79% (Figure 1). We tested the roles of nitric oxide, prostaglandins, and ET‐1 acetylcholine‐induced endothelium‐dependent relaxation using the nitric oxide synthase inhibitor *N*^ω^‐nitro‐l‐arginine methyl ester (l‐NAME), the cyclooxygenase inhibitor meclofenamate, and the ET_A_R antagonist ABT‐627. The maximal relaxation response to acetylcholine was reduced by 54% in the presence of l‐NAME but was not altered significantly by meclofenamate or the selective ET_A_R antagonist ABT‐627 (Figure 1).

**Figure 2.**
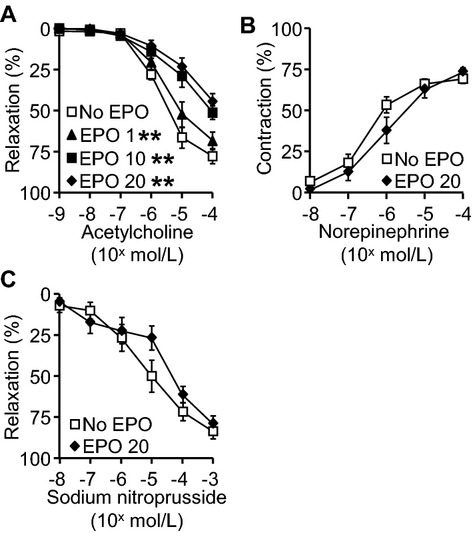
Vascular function of subcutaneous resistance arteries in response to recombinant human erythropoietin (EPO). A, Endothelial function of subcutaneous resistance arteries isolated from chronic kidney disease patients. Vasodilatory concentration‐response curves to acetylcholine were obtained after preincubation (15 minutes) without or with increasing concentration of EPO (1, 10, and 20 IU/mL). Curves were repeated 4 times on the same artery in 10 patients. Concentration‐response curves to acetylcholine without or with 20 IU/mL EPO were performed on the same artery from 7 additional patients. B, Vasoconstrictor response to norepinephrine was not affected by EPO. Vasoconstrictor concentration‐response curves to norepinephrine were obtained after preincubation (15 minutes) without or with 20 IU/mL EPO (n=10 patients, curves performed with and without EPO on the same artery). C, Vasorelaxation response to sodium nitroprusside was not affected by EPO. Vasodilatory concentration‐response curves to sodium nitroprusside were obtained after preincubation (15 minutes) without or with 20 IU/mL EPO (n=8 patients, curves performed on the same artery). Values are means±SEM, *P<0.05 and **P<0.001 vs No EPO.

To determine the effect of EPO, subcutaneous resistance arteries were incubated 15 minutes with EPO and the endothelium‐dependent relaxation response to acetylcholine was determined in presence of EPO. EPO impaired endothelium‐dependent relaxation response to acetylcholine in a concentration‐dependent manner (slope −3.8 AUC unit/EPO unit, *P*=10^−6^). The maximal response to acetylcholine with EPO at 1, 10, and 20 IU/mL was reduced by 12%, 34%, and 43%, respectively, compared with the group without EPO (Figure 2A). EPO had no effect on norepinephrine‐induced contraction (Figure 2B) or on sodium nitroprusside–induced vasorelaxation (Figure 2C).

**Figure 3.**
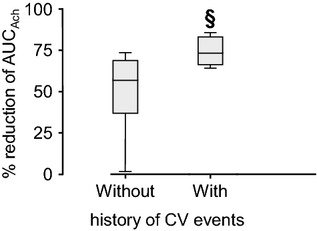
Effect of EPO on endothelial function of resistance arteries isolated from chronic kidney disease patients with (n=4) or without (n=13) history of cardiovascular (CV) events (§*P*<0.05 vs absence of history of CV events (Mann–Whitney *U* test). EPO indicates recombinant human erythropoietin; AUC_Ach_, area under the curve of the relaxation response to acetylcholine.

We next tested the hypothesis that preexisting vascular disease predisposes to the alteration of endothelial function in presence of EPO. In univariate correlation analyses, carotid stiffness was significantly and positively associated with the percent reduction of the area under the curve of the relaxation response to acetylcholine in the presence of EPO (*R*=0.65, *P*=0.01). No association was found with aortic stiffness, carotid intima‐media thickness, or the antihypertensive treatment that patients received. Patients with a history of CV disease had significantly greater reduction in area under the curve of the relaxation response to acetylcholine in the presence of EPO than did the patients without a CV history (Figure 3).

### Mechanisms of EPO‐Induced Small Artery Vasoactive Effects

**Figure 4.**
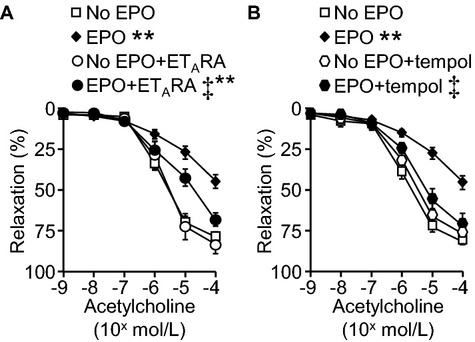
Effects of endothelin (ET)‐1 type A receptor antagonist and reduction of oxidative stress on EPO‐induced endothelial dysfunction. The endothelin type A receptor antagonist (ET_A_RA, A) and tempol, a superoxide dismutase mimetic (B) prevented EPO‐induced endothelial dysfunction. Endothelium‐dependent relaxation response curves of subcutaneous resistance arteries to acetylcholine were obtained after preincubation (15 minutes) without or with 20 IU/mL EPO, without or with 10^−7^ mol/L ABT‐627 in A or 10^−3^ mol/L tempol in (B). Values are means±SEM, *P<0.001 vs Veh, ‡*P*<0.05 vs EPO 20 IU/mL, n=12 patients in (A) and n=15 patients (curves sequentially performed on the same artery for each patient) in (B). EPO indicates recombinant human erythropoietin; ET_A_RA, endothelin‐1 receptor type A blocker ABT‐62.

We tested the ability of the selective ET_A_R antagonist ABT‐627 to prevent the vascular effects of EPO. ABT‐627 reduced the impairment of endothelial function induced by EPO. The maximal relaxation response to acetylcholine with EPO 20 IU/mL was reduced by 44% compared with the group without inhibitor, and in the presence of ABT‐627, the response was reduced by only 14% (2‐way ANOVA, group effect *P*<10^−6^) (Figure 4A). ABT‐627 did not affect endothelium‐dependent relaxation in the absence of EPO.

**Figure 5.**
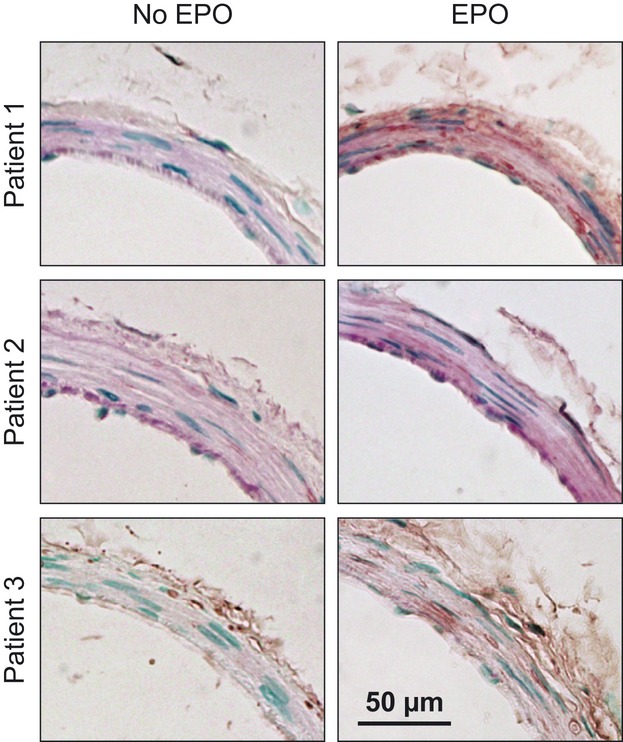
EPO increased the expression of ET‐1 in resistance subcutaneous arteries from chronic kidney disease patients. The expression of ET‐1 was determined by immunohistochemistry in sections of subcutaneous resistance arteries incubated with EPO (20 IU/mL) for 60 minutes. Representative sections of arteries incubated with vehicle (Veh) and EPO are shown. The expression of ET‐1 was determined in sections from 8 patients. EPO indicates recombinant human erythropoietin; ET‐1, endothelin‐1.

**Figure 6.**
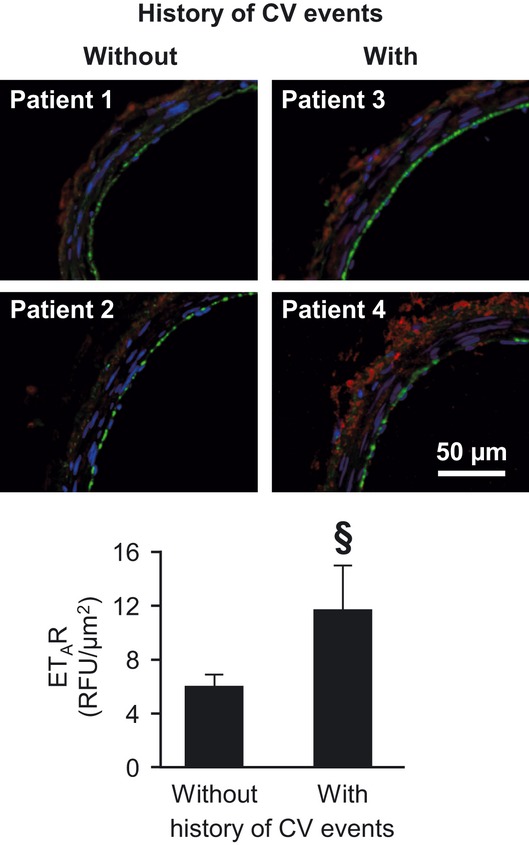
Endothelin type A receptor expression in subcutaneous resistance arteries. Cardiovascular history was associated with greater expression levels of endothelin type A receptor (ET_A_R) in subcutaneous resistance arteries of chronic kidney disease (CKD) patients. The expression of ET_A_R was determined by immunofluorescence in sections of subcutaneous resistance arteries of CKD patients with (n=4) or without (n=13) cardiovascular (CV) history. Representative immunofluorescence sections of arteries of 2 patients per group and the quantification of relative fluorescence (RFU) per surface are shown. Blue, green, and red fluorescence represent, respectively, nuclear 4′,6‐diamidino‐2‐phenylindole (DAPI) staining, autofluorescence of elastin, and ET_A_R staining. Values are means±SEM. §*P*<0.05 vs patients without history of CV events (Mann–Whitney *U* test).

Incubation of small arteries with EPO at 20 IU/mL during 1 hour significantly increased the expression of ET‐1 in the vascular wall (Figure 5). Altogether, these results suggest that the vascular effects of EPO are mediated by ET‐1. In addition, ET_A_R was 2‐fold higher in patients with a history of CV events than in patients without a history of CV events (Figure 6).

We tested the effect of tempol, an antioxidant superoxide dismutase mimetic, on endothelial dysfunction induced by EPO. The maximal relaxation response to acetylcholine with EPO 20 IU/mL was reduced by 45% compared with the group without inhibitor, whereas in the presence of tempol, the response was reduced by only 12% (2‐way ANOVA, group effect *P*<10^−4^) (Figure 4B). Tempol did not affect the endothelium‐dependent relaxation to acetylcholine in the absence of EPO.

## Discussion

In the present study, we demonstrate for the first time that EPO alters endothelial function of small arteries isolated from human subjects with stage 4 CKD, and particularly in patients with a history of CV events. In addition, we provide insight into potential mechanisms by showing that the impairment of endothelial function could be partially prevented by a specific antagonist of ET_A_R and by a superoxide dismutase mimetic. Finally, we show that EPO induced an increase in ET‐1 expression in the wall of resistance arteries.

### Interpretation of the Data

The hypothesis that EPO has a direct effect on vascular function is supported by the observation that the EPO receptor is widely expressed in various tissues, including endothelial cells. The endothelial cell EPO receptor has an intermediate affinity for EPO (≈1 nmol/L), between the affinity of the receptor on erythrocyte precursors (≈200 pmol/L) and the neuronal receptor (≈10 to 20 nmol/L).^28^ Interestingly, the expression of the EPO receptor increases in pathophysiological conditions such as hypoxia.^28^ EPO administration in CKD patients^5^ and in rats^29^ induces an increase in blood pressure that could be related to direct effects of EPO on vascular function or that could be secondary to an increase in hematocrit. In vivo*,* in humans, it is almost impossible to disentangle the direct effect of EPO on the vessel wall from the deleterious effects of increases in hematocrit. Here, we demonstrate ex vivo in human small arteries from CKD patients a direct effect of EPO on endothelial function, which could participate in the increased blood pressure observed in CKD patients treated with EPO.^5^ The results of the present study may explain those of large clinical studies showing that for similar hematocrit levels, patients treated with EPO experience more CV events. For example, in the Reduction of Infarct Expansion and Ventricular Remodeling With Erythropoietin After Large Myocardial Infarction (REVEAL) study involving patients with acute myocardial infarction, a single injection of epoietin alfa (60 000 IU) was associated with poor CV outcomes without differences in hematocrit levels between the control and the treated group.^30^

Several mechanisms could participate in the impairment of endothelial function induced by EPO. EPO increases oxidative stress in the arterial wall.^31^ In human coronary artery endothelial cells, EPO at 5 and 20 IU/mL decreased NO production in response to acetylcholine stimulation, with a parallel reduction in endothelial NO synthase protein abundance.^6^ In agreement, we found that tempol, a superoxide dismutase mimetic, partially prevented endothelial dysfunction induced by EPO. Another potential mechanism is ET‐1 production. Incubation of human umbilical vein endothelial cells with EPO increased ET‐1 generation in a concentration‐dependent manner.^32^ In end‐stage renal disease patients, a significant increase in plasma ET‐1 levels was observed after a single dose of EPO.^33^ The potential role of ET‐1 in EPO‐induced hypertension is underlined by the fact that ET_A_R blockade prevented hypertension induced by EPO administration to uremic rats.^29^ In the present study, incubation of small arteries with EPO induced an increase in ET‐1 expression in the vascular wall. In addition, endothelial dysfunction observed in the presence of EPO was partially prevented by ABT‐627, an ET_A_R antagonist.

Cardiovascular and renal risk factors may influence the alteration of endothelial function found with EPO. A history of CV disease was associated with a greater effect of EPO on the endothelium. Carotid stiffness, which is a marker of CV risk in CKD patients,^34^ was also associated with the alteration in endothelial function with EPO. In addition, our study provides support for the notion that ET‐1 is involved in the vascular effects of EPO. An increased level of ET_A_R was observed in patients with a history of CV events, which may make them more sensitive to an increase in ET‐1 expression. These observations are in agreement with experimental studies showing a major alteration of endothelial function in mice overexpressing ET‐1 treated with EPO compared with wild‐type controls.^35^ In another rodent model, the increase in blood pressure in response to EPO was observed only in uremic rats and not in the control group despite a similar increase in hematocrit.^36^ The fact that the expression of the EPO receptor increases in endothelial cells in pathological conditions such as hypoxia could in part explain these observations.^28^

### Methodological Issues

The strength of this study is that the effect of EPO was studied directly on small resistance arteries from humans with CKD. The tissues used for this study were not surgical samples isolated during a scheduled surgery, which could induce selection bias, but were obtained specifically for this study from gluteal subcutaneous biopsies after informed consent. In addition, this study provides for the first time a possible explanation for the increase in blood pressure and CV events observed in patients with CKD or with coronary heart disease who are treated with EPO.^1–7^ The choice of the population studied was driven by the fact that patients with stage 4 CKD are more likely to receive EPO as part of standard care. In the present study, we believe that the choice of an ex vivo study is an advantage because the effect of EPO on subcutaneous resistance arteries could be dissociated from effects on hematocrit and hemodynamics. Since the goal of this study was to evaluate EPO effects in vivo in the context of CKD, no healthy controls were investigated for comparison, which is a limitation of the study. The concentrations of EPO used in this study were chosen based on published experimental data and could be considered as relatively high concentrations.^6,11,28,32^ However, the maximal concentration measured after 1 intravenous injection of epoietin‐α 150 IU/kg in hemodialysis patients was 2.4 IU/mL.^37^ In addition, EPO was administrated into the chamber, outside the artery, and had to diffuse through the vascular wall. As a consequence, the concentration of EPO that reached the media and the endothelium is likely much lower.

### Perspectives

In the present study, we provide for the first time important insights into mechanisms regarding the effects of EPO on vascular function in humans with stage 4 CKD. These actions are mediated via ET‐1 secretion and oxidative stress. The impairment of endothelial function induced by EPO could contribute to increases in blood pressure and CV events observed in large interventional trials.
